# Probiotics and immunity: provisional role for personalized diets and disease prevention

**DOI:** 10.1186/s13167-015-0036-0

**Published:** 2015-07-14

**Authors:** Rostyslav V. Bubnov, Mykola Ya Spivak, Liudmyla M. Lazarenko, Alojz Bomba, Nadiya V. Boyko

**Affiliations:** Zabolotny Institute of Microbiology and Virology, National Academy of Sciences of Ukraine, 154, Zabolotny St., Kyiv, 03680 Ukraine; Clinical Hospital “Pheophania” of State Affairs Department, Zabolotny Str., 21, Kyiv, 03680 Ukraine; LCL “Diaprof”, Svitlycky Str., 35, Kyiv, 04123 Ukraine; Cassovia Life Sciences, Palárikova 4, 04011 Košice, Slovak Republiс; Institute of Experimental Medicine, Faculty of Medicine, Pavol Jozef Šafárik University in Košice, Trieda SNP 1, 04011 Košice, Slovak Republiс

**Keywords:** Predictive, Preventive, and personalized medicine, Gut microbiota, *Lactobacillus*, *Bifidobacterium*, Gut-brain axis, Microbiome, Cancer, Immune disorders, Dietary biomarkers, Healthy diet, Nutrition, Fecal microbiota transplantation, Pattern recognition receptors

## Abstract

There is great interest in the interaction between diet and immune system and concomitantly in the potential of probiotic bacteria, especially given recent advances in understanding of gut microbiota effects on health in the context of microbiome research. Following our recent study on bacterial wall elasticity as a predictive measure of phagocytic cellular reactions and related outcomes, a question was raised regarding the scope of the application of these findings in various medical conditions in the context of predictive, preventive, and personalized medicine (PPPM). This summarizing review of the data describes the contributions, both observed and potential, of probiotics to the gut-brain axis and various medical conditions, including immune and atopic states, metabolic and inflammatory diseases—including liver disease and diabetes mellitus—cancer, and more. It also suggests novel insights for a number of beneficial applications of probiotics and advances in development of novel probiotic-based treatments and personalized diets, as well as application of sophisticated imaging techniques and nanobiotechnologies that can be adopted in the near future by innovative medical experts, warranting further research and practical translation.

## Review

### Introduction

Healthy diet and nutrition are the very focus of the European Association for Predictive, Preventive and Personalised Medicine (EPMA), the main promoter of predictive, preventive, and personalized medicine (PPPM), and belong to the prioritized medical fields for long-term strategy of created multidisciplinary platform for progressing from “disease care” to “health care”: “advancing participatory medicine”, “well-being” concepts, and integrated approach. A major target of this approach is the effective management of immunity-related disorders, such as inflammation, atopy, asthma, musculoskeletal disorders, liver fibrosis, diabetes mellitus type 2 (T2D), metabolic syndrome, cardiovascular diseases (CVD), neurodegenerative diseases (NDD), atherosclerosis, and cancer [1–4].

The gut microbiota is increasingly considered to be one of the main mechanisms accounting for the increasing prevalence of these disorders over the past few decades. Being a food-grade ingredient, probiotic bacteria show great potential for medical application in general and particularly for personalized, preventive medicine. Lactic acid bacteria (LAB) belong to the group of microorganisms most frequently used as probiotics, due to their competitive inhibition of the colonization of pathogenic bacteria in the intestinal tract and their beneficial effects on the gut immune system and gut-brain axis.

Our recent research employing a non-clinical model investigated the role of cell wall elasticity as a predictive measure of the phagocytic system cells reaction and related outcomes [4]. This review discusses the immediate and far-reaching implications of the findings in the context of the increasing understanding of microbiome’s impact on health.

#### Intestinal microbiota and probiotics for management of immunity-related disorders

The hygiene hypothesis asserts that the increase in the prevalence of atopic disease is related to reduced exposure to microbes at an early life [5–9]. The immunological explanation has been put into the context of the functional T cell subsets known as T helper 1 (TH1) and T helper 2 (TH2) that display polarized cytokine profiles. It has been argued that bacterial and viral infections during early life direct the maturing immune system toward TH1, which counterbalance proallergic responses of TH2 cells. Thus, a reduction in the overall microbial burden will result in weak TH1 imprinting and unrestrained TH2 responses that allow an increase in allergy. This notion is contradicted by observations that the prevalence of TH1 autoimmune diseases is also increasing and that TH2-skewed parasitic worm (*helminth*) infections are not associated with allergy. The elevations of anti-inflammatory cytokines, such as interleukin-10, that occur during long-term helminth infections have been shown to be inversely correlated with allergy [9].

The induction of a robust anti-inflammatory regulatory network by persistent immune challenge offers a unifying explanation for the observed inverse association of many infections with allergic disorders [9].

According to Isolauri and Intestinal Microbiota (NAMI) Research Group report, it was emphasized that “more profound understanding of the complex nature of allergic disorders is needed, as it is likely that there are distinct etiologic factors and pathogenetic mechanisms underlying the heterogeneous manifestations. Second, host-related factors influence the probiotic effects; the distinction in the antiallergic potential of probiotics can be explained by the age of the host and the habitual diet with other potentially active compounds and their conceivable joint probiotic effects” [10].

The allergy is dependent on the balance between TH1 and TH2. According to the study by Shinkai et al. [11], type 2 immunity requires orchestration of innate and adaptive immune responses to protect mucosal sites from pathogens. Dysregulated type 2 responses result in allergy or asthma. TH2 cells elaborate cytokines, such as IL-4, IL-5, IL-9, and IL-13, which work with toxic mediators of innate immune cells to establish environments that are inhospitable to helminth or arthropod invaders. The importance of TH2 cells in coordinating innate immune cells at sites of inflammation is not known. Here, we show that polarized type 2 immune responses are initiated independently of adaptive immunity. In the absence of B and T cells, IL-4-expressing eosinophils were recruited to tissues of mice infected with the helminth *Nippostrongylus brasiliensis*, but eosinophils failed to degranulate. Reconstitution with CD4 T cells promoted accumulation of degranulated IL-4-expressing cells but only if T cells were stimulated with cognate antigen. Degranulation correlated with tissue destruction, which was attenuated if eosinophils were depleted. Helper T cells confer antigen specificity on eosinophil cytotoxicity, but not cytokine responses, defining a novel mechanism that focuses tissue injury at sites of immune challenge [5, 12–14].

There is increasing evidence that the microbiome is able to directly influence the expression of the innate immune system via the toll-like receptor (TLR) pathway [15, 16].

#### Probiotics for immunity-related metabolic conditions

Metabolic disturbances causes a number of diseases, namely, cardiovascular diseases (hypertension, atherosclerosis, coronary heart disease), stroke, insulin-dependent diabetes, premature death, diseases of musculoskeletal system (osteochondrosis and metabolic-dystrophic arthritis), hepatobiliary disease (liver fibrosis, gallbladder dyskinesia, chronic cholecystitis, cholelithiasis), and a number of tumor sites, including lung cancer, breast cancer, uterine, and ovarian cancer.

The strong relationship between obesity and the gut microbiota were demonstrated [17]. Kimura et al. investigating the role of short-chain fatty acid receptor GPR43 and mechanisms linking the gut microbiota with fat accumulation [18].

Recently, probiotic bacteria have been tested for their ability to affect metabolic syndrome. Previously, it was shown that the decrease in the cholesterol concentration in mice with high fat diet caused hypercholesterolemia under the influence of LAB such as *Lactobacillus* and *Bifidobacterium*, in particular *L. acidophilus* ІМВ В-7279, *B. animalis* VKB, and *B. animalis* VKL [19].

The probiotic *Lactobacillus gasseri* SBT2055 was able to reduce adiposity and body weight in obese adults consuming a fermented milk with the bacterium for 12 weeks, potentially by reducing lipid absorption and inflammatory status [20]. A study by Luoto et al. [21] showed the effect of perinatal *Lactobacillus rhamnosus* GG on childhood growth patterns; the probiotic modulated the body weight increase in the early life but had no effect in later stages of development.

The study by Ono et al. demonstrated that *Lactobacillus paracasei* ssp. *paracasei* F19 prevented diet-induced obesity in mice [22]. Lactoferrin (LF), a multifunctional glycoprotein in mammalian milk, is reported to exert a modulatory effect on lipid [23].

Recently, we studied aspects of *malnutrition* and obesity-related effects of probiotic strains [24].

A number of health-promoting properties such as immunological, antimicrobial, antitumoral, and hypocholesterolemic effects have been associated with consumption of fermented milk named “kefir”. Kefir is a good candidate to be used in gut inflammatory disorders [25]. The probiotic treatment with kefir increased IgA in feces and reduced expression of pro-inflammatory mediators in Peyer patches and mesenteric lymph nodes, where it also increased IL-10. In ileum, IL-10, CXCL-1, and mucin 6 genes were upregulated; meanwhile, in colon, mucin 4 was induced whereas IFN-γ, GM-CSF, and IL-1β genes were downregulated. In the case of lactic acid bacteria retrieved from kefir, *Lactobacillus kefiranofaciens* has been proven to ameliorate colitis in a DSS-induced murine model [26] and to produce antiasthmatic effects on ovalbumin allergic asthma mice [27] and the study of the beneficial properties attributed to kefir-isolated microorganisms constitutes a field of great interest for the development of functional foods.

The Lactobacillus and Bifidobacterium strains cannot individually account for all of the effects attributed to different probiotics. The next generation probiotics like *Akkermansia muciniphila* might be suggested for targeting metabolic syndrome [28]. Everard et al. first studied the role of *Akkermansia muciniphila* on host metabolism, mucus layer thickness, glucose and lipid metabolism as well as gut barrier function [29]. The study by Shin et al. confirms that *Akkermansia muciniphila* contribution to improve energy and glucose metabolism [30], and also highlight that metformin treatment increases the abundance of *Akkermansia muciniphila*.

The innate immune system is involved in the pathogenesis of atherosclerosis, acute coronary syndromes, stroke, viral myocarditis, sepsis, ischemia/reperfusion injury, and heart failure [31].

Thus, understanding microbiome activity is essential to the development of future personalized strategies of healthcare, as well as potentially providing new targets for drug development [32].

#### Probiotics for atopy and asthma

Asthma is a common allergic disease and also depends on the imbalance between TH1 and TH2. The inhalation of allergens stimulates both bone marrow-derived cells and non-bone marrow-derived cells in the innate immune system in order to secrete cytokines that promote Ag expressions on CD4+ T cells and activate the APCs and the T cells to produce TH2 responses. TH2 cytokines, such as IL-4, IL-5, IL-9, and IL-13, then induce the changes in the airways and lung parenchyma that are associated with asthma, airway eosinophilia, pulmonary lymphocytosis, mastocytosis, alternative macrophage activation, epithelial cell proliferation with goblet cell hyperplasia (GCH), increased mucus secretion, smooth muscle hyperplasia, hypertrophy, hypercontractility, subepithelial fibrosis, IgE secretion, increased production of chemokines that attract T cells, eosinophiles, neutrophils, mast cells, or their precursors to the lungs, and airway hyperresponsiveness (AHR) [33].

Probiotics improve the balance of intestinal microbes, reduce inflammation, and promote mucosal tolerance. Thus, oral administrations of *L. rhamnosus GG* (LGG) inhibited allergen (ovalbumin or OVA)-induced airway inflammation in a mouse asthma model, decreased matrix metalloproteinase 9 expression in lung tissue, and inhibited inflammatory cell infiltration [34]. Oral treatment with Lcr35 prior to sensitization can attenuate airway inflammation and hyperreactivity in a mouse model of allergic airway inflammation [30]. These results suggest that LGG might had an additional or supplementary therapy for allergic airway diseases anti-inflammatory effect on OVA-induced airway inflammation and may have potential for preventing asthma [34, 35].

Recent data demonstrate the role of *fungal* contamination in asthma genesis. The fungal immunomodulatory protein (FIP-fve) was isolated from *Flammulina velutipes*. Oral FIP-fve had an anti-inflammatory effect on OVA-induced airway inflammations and might posses the potential for alternative therapy for allergic airway diseases [36].

Intestinal microbiota has an important role in signaling to the developing mucosal immune system; effects of probiotics include immunomodulation and restoration of intestinal dysbiosis as well as maintaining epithelial barrier integrity [37]. Both systemic and myeloid tissue-specific A(2B) R deletion significantly decreased pulmonary inflammatory cell recruitment, airway mucin production and pro-inflammatory cytokine secretion after final allergen challenge in sensitized mice. A(2B) R deficiency resulted in a dramatic reduction on TH2-type airway responses with decreased pulmonary eosinophilia without augmenting neutrophilia and decreased lung IL-4, IL-5, and IL-13 production. Pro-inflammatory TNF-α, IFN-γ, and IL-17 secretion were also reduced in systemic and myeloid tissue-specific A(2B) R deletion mouse lines [37]. This TH2-type dysfunction in bronchial asthma inclines to use immunocorrection that can be achieved with probiotics based on *gram*-*positive* microorganisms [7, 38].

Bradykinin receptors in conjunction with increased number of vessels (CD31 cells) and expression of angiogenin and, to a lesser extent, VEGF-A were reported to be overexpressed in the bronchial wall of old patients with asthma in conjunction with an increased expression and fibroblast-derived release of vascular growth factors [39]. Asthma and chronic obstructive pulmonary disease (COPD) are chronic inflammatory diseases of the lung associated with structural vascular remodelling that contributes to airway obstruction. Thus, markers of vascular remodeling, potentially involved in fixed airway obstruction might provide intriguing pathogenesis clues in understanding microcirculation disorders due to vasospasm like *Flammer syndrome* [40] and potential biomarkers for stratification patients within microbiome–gut–circulation–breathing interaction.

The immune-modulatory mechanisms of probiotics on lung disease still deserve further investigation to study the impact on lung diseases in the multidisciplinary scope with consideration of many factors, including fungal infection, vasospasm, lifestyle, smoking, and other forms of tobacco consumption, sport, stress, emotions, psyche, sleep disorders, etc.

#### Probiotics for chronic liver diseases

The knowledge has been cumulated supporting probiotic therapy as a safe, inexpensive, and a noninvasive strategy that can reduce pathophysiological symptoms and improve different types of liver diseases without side effects [41, 42]. Cholestasis is an important diet-related issue for non-alcoholic fatty liver disease (NAFLD) development. Relationship between adipose tissue and fatty liver and its possible evolution in fibrosis, multifactorial pathogenesis of NAFLD, and treatments for various contributory risk factors are well supported by clinical and research experience [43, 44].

Certain probiotics share common beneficial properties, such as improved gut barrier function hepatic bile acid synthesis and reduced hepatic inflammation. The series of experiments showed the mechanisms modulates hepatic bile acid synthesis and metabolism by a mix of probiotics including a description of the FXR-FGF15 axis [45].

#### Probiotics for type 2 diabetes mellitus (T2D)

A large body of evidence proves the effectiveness of probiotics and other therapeutic food additives for T2D patient management. Probiotics reduce the inflammatory response and oxidative stress, as well as increase the expression of adhesion proteins within the intestinal epithelium, reducing intestinal permeability. Such effects increase insulin sensitivity and reduce autoimmune response.

This role of gut microbiota in metabolic diseases opens new prospects in the treatment of obesity, insulin resistance, and type 2 diabetes [46]. One of the features common to metabolic diseases such as obesity and T2D is a mild chronic inflammatory state, which could possibly be—among other factors—the result of TLR activation by lipopolysaccharide (LPS), present in the cell wall of gram-negative bacteria [47]. Probiotic consumption increases the number of bifidobacteria, and increased expression of adhesion proteins reduces intestinal permeability, impairing the activation of TLR4 by bacterial LPS. As result, NFkB activation pathways are blocked. The induction of TH17 cells is also inhibited, preventing pancreatic infiltration of CD8+ T cells [48]. Food additives, probiotics, and prebiotics intake suggested to be new clues, promising for treatment and prevention insulin resistance. Thus, recently, the issues of insulin peroral intake by children at high risk for type 1 diabetes via daily oral administration of 67.5 mg of insulin, compared with placebo, resulted in an immune response without hypoglycemia [49]. However, attempts at both primary preventions, i.e., before seroconversion and secondary prevention, i.e., in those with diabetes-related autoantibodies, have not been successful. The interventions evaluated to date have been limited to those deemed extremely safe because such interventions would be used in at-risk individuals who may or may not actually progress to type 1 diabetes [50].

#### Probiotic role in the gut-brain axis

The *human microbiome project* (HMP) reflects the fact that we are supraorganisms composed of human and microbial components [51, 52].

The conceptual framework for a gut-brain axis has existed for decades. The human microbiome project is responsible for establishing intestinal dysbiosis as a mediator of inflammatory bowel disease, obesity, and neurodevelopmental disorders in adults.

There is increasing evidence that host-microbe interactions play a key role in maintaining homeostasis. Alterations in gut microbial composition is associated with marked changes in behaviors relevant to mood, pain, and cognition, establishing the critical importance of the bi-directional pathway of communication between the microbiota and the brain in health and disease. Dysfunction of the microbiome-brain-gut axis has been implicated in stress-related disorders such as depression, anxiety, and irritable bowel syndrome and neurodevelopmental disorders such as autism [53].

Studies are revealing how variations and changes in the composition of the gut microbiota influence normal physiology and contribute to diseases ranging from inflammation to obesity. Accumulating data now indicates that the gut microbiota also communicates with the CNS—possibly through neural, endocrine, and immune pathways—and thereby influences brain function and behavior [54].

Thus, the concept of a microbiome-brain-gut axis is emerging which suggests that modulation of the gut microflora may be a tractable strategy for developing novel therapeutics for complex stress-related CNS disorders where there is a huge unmet medical need [55, 56].

The notion of the gut-brain axis thereby supports that intestinal microbiota can indirectly harm the brain of preterm infants and initiate neurologic disease in preterm infants. Understanding the anatomy and physiology of the gut-brain axis and transmission of stress signals caused by immune-microbial dysfunction in the gut will offer insight into therapeutic and dietary approaches that may improve the outcomes of very-low-birth-weight infants [57].

#### Probiotics for neuroendocrine applications, APUD cells, serotonin signaling

Neuroendocrine, amine precursor uptake decarboxylase (APUD) cells signaling, serotonin are important and not sufficiently studied mechanisms for a number of pathologies of different localization and link among series of pathological processes as obesity, gut motility, cancer, etc. Serotonin is a primal signaling molecule conserved across phyla that is implicated in the control of energy balance [58–61]. As obesity increases peripheral serotonin, the inhibition of serotonin signaling or its synthesis in adipose tissue may be an effective treatment for obesity and its comorbidities [61]. Crane et al. [61] have found that genetic or chemical inhibition of Tph1 protects or reverses the development of HFD-induced obesity and dysglycemia via activation of UCP1-mediated thermogenesis. Thus, inhibiting Tph1-derived serotonin may be effective in reversing obesity and related clinical disorders such as NAFLD and type 2 diabetes.

#### Probiotics vs. musculoskeletal diseases, pain

The regulatory role of the gut microbiota in immune and inflammatory activity and the metabolic potential that it harbors provide a novel avenue of research for musculoskeletal diseases with potentially novel treatment options [62]. The production of reactive oxygen species (ROS) in different anatomical environments including the GIT by the epithelial lining and the commensal microbe cohort is a regulated process, leading to the formation of hydrogen peroxide which is now well recognized as an essential second messenger required for normal cellular homeostasis and physiological function.

Pain issues should be strongly reconsidered due to role of pain in inflammation, neurodegeneration, and musculoskeletal disorders.

Thus, Fiorentino et al. indicated that joint pathology and pain are dependent on spinal IL-1 and suggested the presence of a bidirectional central nervous system—peripheral joints crosstalk that may contribute to the development, expansion, and exacerbation of arthritis [63]. Pain should no longer be thought of as just a symptom of arthritis; pain signals originating in arthritic joints and the biochemical processing of those signals as they reach the spinal cord worsen and expand arthritis; nerve pathways carrying pain signals transfer inflammation from arthritic joints to the spine and back again, causing disease at both ends. Thus, it might be speculated that “not only does arthritis cause pain but pain causes arthritis”. Continuous control of pain and inflammation which will increase the functionality of the arthritic affected joints is the essential pathogenic treatment with pain management in focus.

Novel integrated concept for pain management in the consolidated paradigm of PPPM [64] should by implemented in the highest levels of multidisciplinarity and benefits from excellent competencies of all medical fields (including neuroscience, psychology, imaging, sport medicine, and TCAM) and complex technological instruments (including hybrid technologies). The main focus is a deep diagnostics followed by creating individualized treatment algorithms, involving experts in a variety of medical specialties, promoting innovations in rehabilitation, physiotherapy, neuromuscular treatment, military medicine, and regenerative medicine [65]. Sophisticated cytokine pattern and image-guided individualized therapy as *platelets rich plasma* (PRP) under ultrasound guidance [66] is an example of effective *individualized* hybrid technique.

Gut-brain axis might be hypothesized as pain reducing mechanism via regulating nerve and muscle inflammation, and thus its function, and affecting central sensitization.

Recently, the has also been observed the growing body of evidence linking the microbiome with rheumatoid arthritis (RA) pathogenesis [67–69], however, still far from a novel concept [70].

Changing diets, altering the gut microbiota towards dysbiosis has been hypothesized to be driving an increase in the incidence of inflammatory diseases [5]. Obesity is regarded as a chronic low-grade inflammatory state, and inflammatory cytokines secreted from adipose tissue are associated with RA [69, 71].

Animal models set a solid data for linking bacterial antigenicity to the generation of inflammatory arthritis [69].

Recent study used molecular biological techniques to compare the faecal microbiota of patients with fibromyalgia and RA [67]. RA patients had significantly reduced faecal carriage of common commensals including bifidobacteria and *Bacteroides fragilis*. Mucosal sites exposed to a high load of bacterial antigens and autoimmune generation - such as the periodontium [72], pulmonary parenchyma [73], and gut – were suggested as possible mucosal sites of initiation of autoimmunity in RA. Taken together, in vitro, animal, and human data, advanced imaging techniques should be validated to the discover potential biomarkers and therapeutic approaches in the pre-clinical and clinical phases of RA.

It is essential to make efforts in increasing the level of evidence of personalized procedures performing the studies of development of pain biomarkers for predictive approach and for measuring outcomes, self-assessment, development of relevant questionnaires for participating medicine, and studies of mutual impact of pain, lifestyle, metabolism, nutrition, gut-brain axis (GBA) for prevention of wide spectrum of collateral diseases (NDD, metabolic syndrome, cancer), and suggesting new health care policy, smart decision-making, and advances in education for economic benefits for aging society and working population.

#### Probiotics vs. cancer

Various mechanisms that elucidate the preventive role of probiotics in colon cancer risks discussed in various in vitro and in vivo based studies indicate that the use of probiotics may prevent the risk of colon cancer [4, 74–76]. But most of the studies related to prevention of colon cancer by using probiotics are unclear; further confirmation studies are needed, and the observed effects cannot be generalized. Future research is needed in terms of the underlying mechanism of action involved in each of the observed effects [77].

One of the most promising areas for the development of *functional foods* lies in modification of the activity of the gastrointestinal tract by use of probiotics, prebiotics, and *synbiotics*. A myriad of healthful effects have been attributed to the probiotic lactic acid bacteria; perhaps, the most controversial remains that of anticancer activity. There is no direct experimental evidence for cancer suppression in humans as a result of consumption of lactic cultures in fermented or unfermented dairy products [77].

It was reported that administration of bifidobacteria or lactobacilli alone or with fermentable carbohydrate (defined as a prebiotic) can alter colonic microflora populations and decrease the development of early preneoplastic lesions and tumors [78]. The new knowledge is essential to be developed regarding the effect of gut microbiota on common cellular and molecular mechanisms in the pathogenesis of cancer, atherosclerosis, and comorbidities and the possibilities of its modulation by probiotics and prebiotics in their prevention [4, 79, 80].

#### Gender-specific needs for diet

Gender-specific integrated *Women* and *Men health* concepts have been extensively assessed within the large scope of factors affecting fertility and general health, which connect to lifestyle, diet, obesity, and gender-related pathology [81, 82].

Vaginal and male genital tract ecosystems as the functional interaction between the genital microbiota and the host, and the association of semen and vaginal microbiomes are still poorly studied [83]. Thus, neither clinical criteria, nor community composition and structure can fully explain symptomatic bacterial vaginosis. *Lactobacillus* species dominate vaginal microbiota in the majority of normal and healthy women [84], *Gardnerella vaginalis* was predominant in half of the women whose partners had significant leukocytospermia [83]. Current knowledge of the male genitalia microbiome is very limited. Studies of structure of vaginal microbiota in regards to inflammatory conditions via analysis of samples collected in the various stages of disease and in different at-risk populations, in regards to the role of host genotype, involvement hormonal receptors might suggest promising approach for understanding pathogenesis of chronic gender-related inflammatory diseases, development personalized treatments, diet and lifestyle corrections.

#### Probiotics: treatment approaches to balance metabolome

Microbiome correction via fecal microbiota transplantation (FMT) is considered to be effective method for treatment of *Clostridium difficile* infection (CDI) and also for recurrent CDI.

Fecal transplantation through colonoscopy appears to be an effective treatment for recurrent CDI caused by the virulent *C. difficile* 027 strain [85, 86].

FMT is recommended to hospitals and should encourage the development of fecal transplantation programs to improve therapy of local patients for the treatment of recurrent CDI [87].

FMT has proven to be an effective and safe procedure for the treatment, also for immunocompetent patients by restoring the gut microbiota [88].

Stable cure can be achieved by restoring the gut microbiome with an effective, well-tolerated microbiota transfer via orally administered capsule treatment [89].

This strategy of microbiota transfer can be widely applied and is particularly appropriate for frail patients.

However, the method still lacks evidence, also for extracolonic pathology, as in the randomized controlled trial of fecal transplantation efficacy for patients with ulcerative colitis (phase 2 trial) by Rossen et al. [90]. There was no statistically significant difference found in clinical and endoscopic remission between patients with ulcerative colitis who received fecal transplants from healthy donors and those who received their own fecal microbiota, which may be due to limited numbers.

Moreover, FMT does not seem to provide the same safety profile showed for non-IBD individuals with *C. difficile* infection. The available evidence is limited and weak. FMT has the potential to be somehow of help in managing patients with IBD, but considerable further efforts are necessary to make this procedure a valid option for these subjects [91].

#### Probiotics: part of a healthy diet

Food production, human nutrition, and the incidence of diet-related diseases are becoming increasingly important in our rapidly changing scientific, economic, and societal environments. High quality diets and proper physical activity are the most critical determinants in human health and for the quality of life in an aging society [92].

Significant number of Horizon 2020 calls [93] are oriented to nutrition and in particular to the implementation of a Joint Programming Initiative on “A Healthy Diet for a Healthy Life”, to coordinate research on the impact of diet and lifestyles on health, significantly contributing to the construction of a fully operational European Research Area for the prevention of diet-related diseases and strengthening the leadership and competitiveness of research activities in this field. A Healthy Diet for a Healthy Life (JPI HDHL) claims ambitious vision [94]: “By 2030 all Europeans will have the motivation, ability and opportunity to consume a healthy diet from a variety of foods and have healthy levels of physical activity, and that the incidence of diet- related diseases will have decreased significantly.”

The consumption of probiotics has to be studied and implemented with strong agreement to beneficial and functional foods patterns, performed in the personalized approach, predicted by relevant properly applied and interpreted *dietary biomarkers*, evidence of gene-probiotic-nutrient interactions [95] assessed with proper data collection tools such as food frequency questionnaires (FFQ) [96], collecting samples of biologic materials (fecal, saliva) [97], and proved to prevent many diseases. For instance, fecal bacterial DNA samples collected during the clinical in randomized controlled trial the novel rigorous information was obtained demonstrating the impact of dietary fiber supplementation inducing changes in the gastrointestinal microbiome of healthy adults [97]. Thus, smart collection of such personalized data requires development of electronic devices, gadgets for electronic patient profiling, and could be crucial for fast feedback based on individualized diet planning in the near future.

On the other hand, the modern nutritional recommendation originally inspired by globally acknowledged health dietary patterns as *UNESCO* recognized Intangible Cultural Heritage diet pattern of Mediterranean diet [98], effectively consolidated with national diet patterns as, e.g., Black Sea countries diet [99], should be suggested and implemented and popularized in order to elucidate their role in the dietary pattern of populations and to preserve and promote these foods to meet the expected impact announced in FOOD Horizon 2020 calls [100] and support to the research and innovation activities carried out in the European Innovation Partnership on Active and Healthy Ageing to develop “innovative products and services for the elderly” and for “better understanding of the interaction between nutrition and the aging process through exchange of knowledge/best practice” [100].

#### Future prospects to increase the evidence of effective treatments

Nevertheless, the use of probiotics has several drawbacks, namely, introduction of foreign microorganisms induces antagonistic activity against pathogenic and indigenous microorganisms and eliminates fast the probiotic strain. Therefore, to achieve the personalized approach, the development and application of products manufactured from own strains of organism seem promising. For this reason, certain individual microorganisms might be grown on artificial nutrient, exploring their environment friendliness, establishing spectrum antagonistic effects on the body. The potential alternative for probiotics might be the suggested *lysates* of probiotic strains that also exhibit immune-modulatory activity [24].

*Prebiotics* have large potential to enhance probiotics effects. A prebiotic was first defined by Gibson et al. as a “non-digestible food ingredient that beneficially affects the host by selectively stimulating the growth and/or activity of one or a limited number of bacteria in the colon, and thus improves host health” [101]. The key point is the specificity of microbial changes [102]. Being selectively fermented, food ingredients that induce specific changes in the composition and/or activity in the gastrointestinal microbiota prebiotics are beneficial to the host’s well-being and health have a protective effect and may be useful for many conditions like colon cancer prevention and treatment [103]. Thus, herbal-based biopolymers as *fenugreek* having antiobesogenic properties might offer effective added advantages as prebiotic towards the enhancement of probiotic bacterial growth in the gastrointestinal environment [104].

Interesting and promising is the combined use of probiotics with nanoparticle-based treatment and food supplements. Thus, nanoparticles of gold [105, 106] and cerium dioxide [107, 108] were reported as strong agents against oxidative damage having anti-aging activity. Nanoparticles of cerium dioxide, considering its UV-shielding effect, antiviral, antibacterial, antifungal activity, cardioprotective, neurotrophic, hepato- and nephroprotective, and anti-aging effect, have potential for various biomedical applications [107]. Treatment with nanoceria has also supplementary perspectives in reproductive medicine, enhancing female [108] and male fertility [109]. We recently reported effects of CeO_2_ nanoparticles affecting gastrointestinal motility on rat model and reviewed data supporting their perspectives to be applied as effective laxatives [110].

The low-dose cytokine prescription for correction of different disorders is an essential part of personalized diet and pharmacology in Medicine of the Future as, e.g., concepts of Physiological Regulating Medicine (PRM), which led to development of novel drugs like *GUNA*’*s* products [111].

However, many novel treatment techniques in spite they show their effectiveness still lack rigorous scientific support (level-I evidence); that means that at least randomized controlled trials have been performed, and the treatment approach has been found to be effective. However, today, many of usual every day practice treatments are still not supported by level-I evidence. Thus, many orthopedic procedures (includes joint arthroscopy, microfracture surgery), spinal surgeries (including fusion, laminectomy, and discectomy), chiropractic adjustments, acupuncture, massage, and most of physical therapy and complementary and alternative medicine (CAM) [65].

For this reason, considering discussed above, future research planning should be focused to increase the level of evidence for promising and effective techniques (like probiotics, prebiotics, nutritional, biological therapies, etc.) that is still not sufficiently supported by evidence and adhere study protocols to the PPP medicine, including (1) in vitro studies, (2) in vivo studies in laboratory animals, (3) epidemiological studies, and (4) studies in human volunteers and patients with relevant assessment of efficacy of each particular case.

This integrated vision is a logic development of the concepts foreseen by Ukrainian scientist Elie Metchnikoff, the founder of concepts of probiotics, phagocytosis, and gerontology, etc., the Nobel prize winner in 1908, who studied aging and longevity, created a theory of aging caused by toxic bacteria emerging, had developed the “optimistic conception of prolongation of human life” [112] that becomes to a reality today.

## Conclusions

### Consolidation of the PPPM concept

Based on this review of the data, a number of beneficial application of probiotics and advances in development novel probiotic-based treatments and personalized diets can be suggested based on sophisticated imaging techniques and nanobiotechnologies that can be adopted in the near future by innovative medical experts, warranting further research.

### Personalized medical approach

Designing person-related probiotic strains and smart physiologic low-dose treatments in order to correction the gut microbiome is an important impact to personalized dietology and is a challenge for *medicine of the future*. As the microbiome is a complex ecosystem, it is challenging to conduct clinical studies for stratifications of potential responders to specific strains to formulate clear personalized application protocols, considering underlying mechanisms governing the cross-talk between individual bacterial strains and a host. So far, cumulated evidence allows to provide interventions with high level of personalization. Considering biosafety of probiotics-based products, the group of potential patients (consumers) can be large. To achieve the personalized approach, the development and application products manufactured from own strains of organism are promising. On the other hand development a realistic vision is important to minimize potential risks avoiding considering gut microbiota alone as a panacea, that is possible via integrated and balanced PPPM approach.

### Predictive medical approach

Translating obtained data on animal model to human organism allows to consider consumption of the products under guidance of patient’s profile data including relevant disease and dietary related biomarkers.

Development of the panel of biomarkers to recognize specific patterns of immunity-related diseases in musculoskeletal, respiratory, metabolic syndrome, CVD, NDD, and cancer from the point of view of extensive vision including gut-brain axis (GBA), intestine microbiota, psyche, stress, emotions, pain, physical activity, and molecular and cellular mechanisms is an important point to develop targeted intervention and preventive measures with probiotics, prebiotic, and synbiotics.

Metabolic profiling, including gut microbiota, is a crucial part of the platform for development and validation, sophisticated application, and interpretation of biomarkers for nutrition and health, including biomarkers for food intake and for the risk of diet-related disease for the inclusive of a more holistic approach assessing gene-probiotic-diet-lifestyle-disease associations.

Development of sophisticated non-invasive imaging *in vivo* techniques of microbiome and microbe-host interaction, including *magnetic resonance imaging (MRI), ultrasound*, and *positron emission tomography (PET), endoscopy*, *microscopy* data as *AFM* may support additional information regarding colonic microbiota and the colonic mucosa, allow the determination of both the location and the number of infection and inflammatory foci in virtually all tissues, providing imaging biomarkers, that we discussed in [4].

Synergistic *in vivo* and *in vitro* “*pan*-*microbiome*” studies in various mucosal sites, including extra-colonic microbiome in the oral cavity, nasopharynx, respiratory tract, genital tract, bladder, skin, and also a vascular microbiome (as blood vessels may not be sterile and vascular microbial communities are related to noninflammatory and inflammatory vessel diseases [113]), microbiome in childhood, ageing and pregnancy [114], particularly in genetically predisposed individuals and tailored for each gender is promising for discovery potential biomarkers of chronic diseases and may provide important insights into disease pathogenesis and suggest new therapies.

The main focus on biomarkers for collateral immune-related disorders, proper stratification of patients, and biological treatments according to growing evidence in the field is the prediction of strain efficacy according to their properties (including AFM data) and treatment outcome for different strains.

Potential sources of biomarkers are the parameters of bacterial strain cell walls (as cell wall elasticity, receptor status, probiotic cell molecular patterns, etc.) and the patterns of cytokine induction in the macrophages culture. It is necessary to use well-defined evaluation procedures for the identified biomarker to ultimately allow the clinical validation [115]. A panel of “immunity assessment for personalized diet biomarkers” should include *patterns of cytokine induction*; *oxidative stress* biomarkers: DNA oxidation biomarkers (oxidized DNA bases such as 8-OHdG, autoantibodies to oxidized DNA, modified comet assay), lipid oxidation (thiobarbituric acid-reactive substances, exhaled pentane/ethane, low-density lipoprotein resistance to oxidation, isoprostanes), and protein oxidation (protein carbonyls); *Nitrosative stress* biomarkers: NO, nitrite, peroxynitrite, and inducible NO synthase (iNOS) expression, modificated proteins, nitrosothiols, 8-nitroguanine (a marker of nitrative DNA damage), etc.; *Apoptosis* biomarkers (Ki67, etc.); and *Vasospasm* biomarkers (endothelin, NO, etc.) [40].

### Preventive medical approach

We suggest that LAB and bifidobacteria and novel strains might be an additional or supplementary therapy and may have potential for preventing wide scope of immunity-related diseases due anti-inflammatory effect. The next generation probiotics strains should be properly studied and suggested to clinical application. Translation of the obtained data on animal model to human organism may allow to consider diet correction with probiotics for balancing immunity, in particular in elderly for promotion of health based on smart patient profiling with relevant gut microbiome data and immune response patterns guided physiologic diet and lifestyle in the integrated vision of an interactome.

## Abbreviations

AFM: atomic force microscopy
CVD: cardiovascular diseases
DCs: dendritic Cells
DGGE: denaturing gradient gel electrophoresis
EPS: exopolysaccharides
FMT: fecal microbiota transplantation
GBA: gut-brain axis
LAB: lactic acid bacteria
LPS: lipopolysaccharide
NDD: neurodegenerative diseases
NLRs: NOD-like receptors/nucleotide-binding oligomerization domain receptors
PG: peptidoglycan
PPPM: predictive, preventive, and personalized medicine
PRRs: pattern recognition receptors
T2D: diabetes mellitus type 2
TLR: toll-like receptors
